# Interleukin-21 Drives Proliferation and Differentiation of Porcine Memory B Cells into Antibody Secreting Cells

**DOI:** 10.1371/journal.pone.0171171

**Published:** 2017-01-26

**Authors:** Michael C. Rahe, Michael P. Murtaugh

**Affiliations:** Department of Veterinary and Biomedical Sciences, University of Minnesota, St. Paul, MN, United States of America; The Ohio State University, UNITED STATES

## Abstract

Immunological prevention of infectious disease, especially viral, is based on antigen-specific long-lived memory B cells. To test for cellular proliferation and differentiation factors in swine, an outbred model for humans, CD21^+^ B cells were activated *in vitro* with CD40L and stimulated with purported stimulatory cytokines to characterize functional responses. IL-21 induced a 3-fold expansion in total cell numbers with roughly 15% of all B cells differentiating to IgM or IgG antibody secreting cells (ASCs.) However, even with robust proliferation, cellular viability rapidly deteriorated. Therefore, a proliferation inducing ligand (APRIL) and B cell activating factor (BAFF) were evaluated as survival and maintenance factors. BAFF was effective at enhancing the viability of mature B cells as well as ASCs, while APRIL was only effective for ASCs. Both cytokines increased approximately two-fold the amount of IgM and IgG which was secreted by IL-21 differentiated ASCs. Mature B cells from porcine reproductive and respiratory virus (PRRSV) immune and naïve age-matched pigs were activated and treated with IL-21 and then tested for memory cell differentiation using a PRRSV non-structural protein 7 ELISPOT and ELISA. PRRSV immune pigs were positive on both ELISPOT and ELISA while naïve animals were negative on both assays. These results highlight the IL-21-driven expansion and differentiation of memory B cells *in vitro* without stimulation of the surface immunoglobulin receptor complex, as well as the establishment of a defined memory B cell culture system for characterization of vaccine responses in outbred animals.

## Introduction

The memory B cell is a critical component of protective long-term immunity against reinfection. Following antigenic recognition, its ability to rapidly proliferate and differentiate into antibody secreting cells (ASC) results in the production of antigen-specific antibodies. These antibodies are essential for binding and clearance of invading pathogens prior to the incidence of clinical disease. Previous *in vitro* work in the pig has shown that this secondary humoral immune response requires antigen specific T cell help [1, 2]. However, the factors necessary to stimulate robust porcine B cell expansion and differentiation to ASCs have not been extensively studied, except in a mixed leukocyte culture system [3, 4]. Work on human and mouse B cells has shown that, while many cytokines are capable of producing a proliferative and differentiating response, IL-21 is the most potent at driving this response [5].

Interleukin-21 (IL-21) plays a key role in B cell biology, including the ability to robustly proliferate and differentiate activated naïve, germinal center, and memory B cells [2, 6–8]. It also has implications in pathological sequelae in the development of autoimmunity, rheumatoid arthritis, and transplant rejection [9–11]. Collectively, this work has resulted in an enhanced understanding of how the adaptive immune system responds to antigenic recognition while also shedding light on the pro-inflammatory effects of IL-21. However, all previous research on IL-21 function has been limited to the mouse and human, resulting in a gap in knowledge of the function of IL-21 in outbred animal models including animals which are important for nutrition, food and fiber.

The pig is a critical model species for biomedical research in diabetes and islet transplantation while at the same time is susceptible to a multitude of pathogens for which the memory immune response has not been characterized [12]. The use of the pig for research and the ability to develop vaccines which stimulate an effective memory response have previously been hindered by a limited understanding of the factors which drive B cell differentiation. To date, the role of IL-21 in the pig adaptive immune response has not been investigated. Failure to understand the function of IL-21 on the pig B cell has prevented development of *ex vivo* strategies for evaluating protective memory responses to devastating pathogens, such as porcine reproductive and respiratory syndrome virus (PRRSV) a rapidly mutating RNA virus. Furthermore, a deficient understanding of the roles of important cytokines in porcine B cell biology has obstructed advances in the translational study of diabetes and transplantation immunology.

Here, we investigated the effects of IL-21, along with several other cytokines and factors (CD40L, IL-4, BAFF, APRIL) on CD21-positive porcine B cells. CD21 was used as a B cell marker due to its expression on all mature B cells, including memory B cells [13]. These studies utilized an *in vitro* system to evaluate the effect of cytokines on mature B cell activation, proliferation, viability, and differentiation to ASCs. Finally, IL-21 was evaluated for its ability to proliferate and then differentiate PRRSV non-structural protein 7 (nsp7) specific memory B cells into antigen-specific ASCs. Our results demonstrate the proliferative and differentiating effects of IL-21 in porcine B cells, reveal the roles of BAFF and APRIL for inhibiting porcine ASC apoptosis and maintaining cellular viability, and confirm a previous finding of a species-dependent difference of the B cell stimulatory effect of IL-4. It is now possible to establish optimal culture conditions for the expansion, differentiation, and evaluation of porcine memory B cells to specific antigens that can inform the role memory B cells in controlling specific diseases and serve as an outbred model for human immune responses to infectious diseases.

## Materials and Methods

### Antibodies and reagents

The following antibodies were used: unlabeled goat anti-pig IgM and IgG polyclonal antibodies (Ab) (Bethyl), mouse anti-porcine IgM monoclonal antibody (mAb) (AbD Serotec K52 1C3), hamster anti-mouse CD80 PE-Cy7 mAb, mouse anti-pig SLA Class II DR mAb (AbD Serotec 2E9/13), horseradish peroxidase (HRP)-conjugated goat anti-pig IgM and IgG polyclonal Ab (Bethyl); phycoerythrin (PE)-labeled mouse anti-porcine CD21 mAb (Acris Antibodies BB6-11C9.6); FITC-goat anti-pig IgG polyclonal Ab (Bethyl); and eFluor 780-Fixable Viability Dye (eBioscience). Unlabeled antibodies were conjugated with the following APEX antibody labeling kits: Alexa Fluor 488, Alexa Fluor 568, Alexa Fluor 647, and Pacific Blue. Reagents used for tissue culture included recombinant human (rh) BAFF and APRIL (Peprotech); IL-4 (Gibco); CD40L (BioLegend), and IL-21 (eBioscience). Cells were cultured in complete RPMI 1640 with L-glutamine, 10% fetal bovine serum (FBS), 10 mM HEPES pH 7.2, 1% non-essential amino acids, 1% sodium pyruvate, and 20 μg/ml gentamycin.

### Isolation and culture of CD21+ porcine B cells

Spleens from naïve or PRRSV-infected pigs were procured via tissue sharing with other investigators at the University of Minnesota. Dissociation of splenocytes from structural tissue was achieved by forceful pressing of splenic tissue pieces through a metal screen into culture media. Resulting cells and media were collected and passed over a 40 um filter. Cells were pelleted gently, and treated with ACK lysis buffer (Lonza) to remove red blood cells. Remaining cells were washed with ice cold PBS and retreated with lysis buffer. Cells were washed again, counted, put into freezing media (50% FBS, 40% complete RPMI 1640 media, and 10% DMSO) at ~10 million cells/ml, and frozen in liquid nitrogen following several cooling steps.

For experiments, cells were thawed from liquid nitrogen and magnetically enriched as described by Crawley et al. [4]. Briefly, cells were labeled with mouse anti-porcine CD21-PE. Miltenyi-Biotec mouse anti-PE microbeads were added and the suspensions were passed over consecutive Miltenyi-Biotec LS columns. CD21+ enriched cells were suspended in culture media, counted, assessed for viability and enrichment (>98%), and then placed into 96-well round bottom non-adhesive culture plates (Sarstedt) for 7 days at 50,000 cells/well. Cytokines, when used, were included at the following concentrations: CD40L (1.0 ug/ml), IL-4 (50 ng/ml), IL-21 (50 ng/ml), BAFF (100 ng/ml), APRIL (100 ng/ml). Concentrations were based off of previous literature and optimized for biological activity in our *in vitro* system [14–19].

### Flow cytometry

LSR Fortessa (BD Biosciences) and LSRII (BD Biosciences) instruments were used for flow cytometry. FACSDiva Software (BD Biosciences) was used for data acquisition. Data analysis was performed with FlowJo Software V10 (Tree Star).

### B cell proliferation analysis

Cellular divisions were assessed by tracking the dilution of intracellular CFSE. Purified CD21^+^ B cells were labeled with CFSE and cultured in triplicate either alone, with CD40L, or with CD40L and test cytokines. Cells were analyzed for proliferation and viability at 7 days of culture by flow cytometry. Total cell numbers for each condition were assessed in triplicate each day using trypan blue staining and a hemocytometer.

### Intracellular Ig and annexin V staining

Cell cultures were treated with 1X Brefeldin A Solution for one hour, then taken out of culture and resuspended in MACS buffer. Fixable viability dye (FVD) and Pacific blue labeled anti-IgM and IgG antibodies were added to the staining cocktail in order to coat and detect surface immunoglobulin. These cells were then fixed and permeabilized with a Cytofix/Cytoperm kit (BD). Following fixation and permeation, cells were washed with Cytofix/Cytoperm wash buffer. The same anti-IgM and IgG antibodies that were used in the previous step, but conjugated to APC, were then used to stain intracellular immunoglobulin. Finally, cells were washed twice with Cytofix/Cytoperm wash buffer and evaluated via flow cytometry.

For Annexin V staining, cells were stained with the same extracellular antibodies and FVD as previously described for extracellular staining. Following EC staining, cells were washed with PBS, resuspended in 1X Annexin V binding buffer (FITC Annexin V apoptosis detection kit I, BD), and stained with Annexin V according to the manufacturer’s protocol. Cells were washed with 1X binding buffer and resuspended in Cytofix/Cytoperm supplemented with 12.5 mM CaCL_2_ [20]. 10X Cytofix/Cytoperm wash buffer was then diluted to 1X in Annexin V binding buffer and used for all subsequent washes and remaining IC staining.

### Quantification of IgM and IgG secretion

Total secreted IgM and IgG in culture supernatants were assessed using a standard sandwich ELISA with goat anti-porcine IgM and IgG unlabeled polyclonal Abs to bind isotype specific immunoglobulins. Horseradish peroxidase conjugated goat anti-pig IgM and IgG antibodies were used to detect the presence of bound Ab. ELISA plates were read with a Bio-Tek plate reader at OD450 and analyzed with Gen5 2.05 software (Bio-Tek).

### ELISPOT assay

ELISPOT was performed as described with the following modifications and using porcine reagents [3, 21]. Plates were coated with 0.5 ug/well of nsp7 or anti-pig IgG polyclonal antibody, and HRP-conjugated anti-pig IgG polyclonal antibody was used as the detection reagent. Also, following AEC addition and washing, the back of the ELISPOT plate was removed and the backsides of the membranes were washed with tap water to remove unbound dye and eliminate background staining of the membrane.

## Results

### CD40L as an activator of porcine B cells

**Fig 1:**
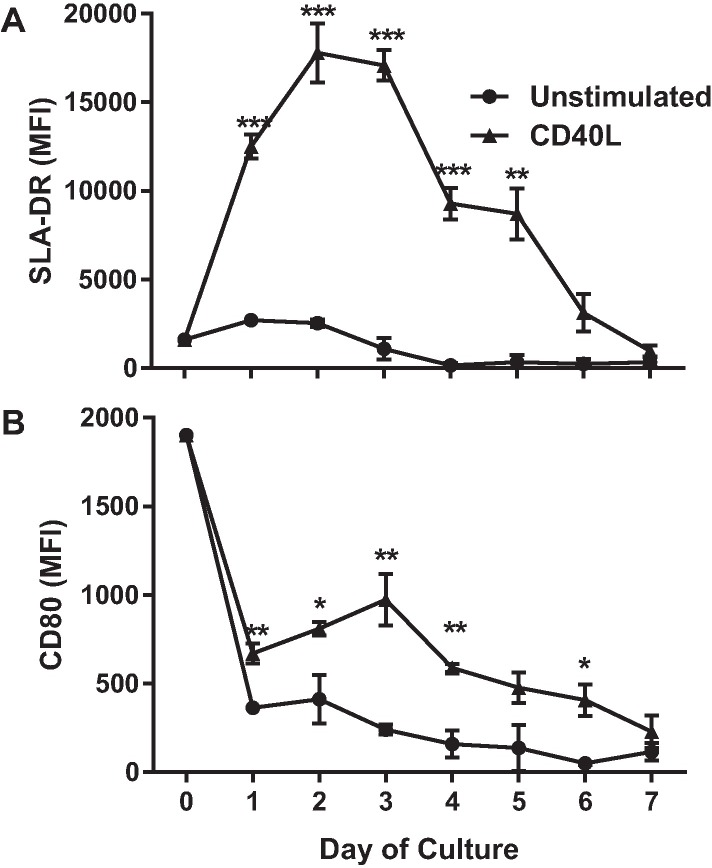
CD40L induces B cell activation. CD21^+^ purified B cell populations were cultured in the presence or absence of CD40L (1.0 ug/ml), gated on live cells, and then analyzed for changes in the median fluorescence intensity (MFI) of SLA-DR (A) and CD80 (B). Cells were analyzed prior to culture and at 24 h intervals for a period of 7 days. One representative experiment of two performed is displayed. Data shown are mean ± SEM. Statistical differences of *p ≤ 0.05, ** *p* ≤ 0.01, ***p ≤ 0.001 are indicated.

To investigate the ability of CD40L to activate porcine B cells we magnetically enriched CD21^+^ splenocytes and cultured them for one week in the presence or absence of soluble human CD40L (CD154). Human CD40L binds porcine CD40 on pig endothelial cells and induces biological activity [22, 23]. Expression of CD80 and MHCII was evaluated using flow cytometry every 24 hours on both CD40L treated and untreated cells. Both CD80 and MHCII have previously been used as activation markers for mouse, human and porcine B cells [24–28]. CD40L treatment resulted in a seven-fold increase in MFI of MHCII expression by day 2 of culture. This significant difference in MHCII expression between treated and untreated cells gradually declined to levels observed on live unstimulated cells by day 6 (Fig 1A). CD80 expression was drastically reduced on all B cells when put into culture. However, live cells that were treated with CD40L maintained higher levels of CD80 expression for the first 6 days of culture (Fig 1B).

### B cell activation is necessary for robust IL-21 stimulation

Previous work with porcine B cells showed that cellular activation with CD40L induced low levels of proliferation in certain subsets of B cells and that activation was necessary for many stimulatory cytokines to elicit an effect [28]. During initial cultures, comparing the activation of CD21^+^ cells, we also noticed a difference in cellular proliferation between CD40L-activated and non-activated cultures. Therefore, we monitored proliferation over 7 days of culture after labeling cells with CFSE. Additionally, we tested the effect of two purported B-cell stimulatory cytokines, IL-4 and IL-21, on porcine B cells to determine whether activation was necessary for stimulatory cytokines to elicit a proliferative and differentiating response.

**Fig 2:**
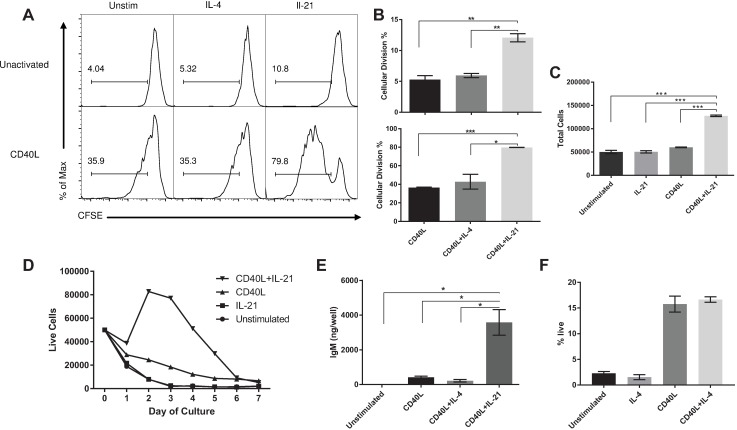
IL-21 is a potent inducer of CD40L-activated B cell proliferation and differentiation. Quiescent or CD40L activated CD21^+^ cells were stimulated with IL-4 or IL-21 at 50 ng/ml. (A) CD21^+^ cells were stained with CFSE and analyzed by flow cytometry at day 7 of culture for proliferation. (B) Proliferating percentages for each treatment condition. (C) Total cell numbers after 7 days of culture. (D) Cell culture viability dynamics. (E) IgM production from activated and stimulated cells, quantified with a total IgM ELISA of culture supernatant. (F) Cell viability evaluated by fixable viability dye staining and flow cytometry at day 7. One representative experiment of three with three different animals is displayed. Data are mean ± SEM. Statistical differences of *p < 0.05, ** p < 0.01, ***p < 0.001 were determined using ANOVA to compare group means followed by t-tests to calculate significance.

In the absence of CD40L, IL21 induced a low level (~10%) of proliferation, whereas IL-4 showed no activity (Fig 2A and 2B). Following CD40L activation, IL-21 induced a potent proliferative response and an approximately 2.5-fold increase in total cell number (Fig 2B and 2C). Most cellular divisions occurred between 24 and 72 hours of culture evidenced by the sharp rise in live cells during this time period (Fig 2D). These cells appeared to be short lived, as there were few viable cells left in culture by day 7. IL-4 displayed no proliferative effect in the presence of CD40L activation (Fig 2B).

IL-21 has been reported in the human and mouse to be important for B cell differentiation into antibody secreting cells [2, 29, 30]. After 7 days of porcine B cell culture in the absence of CD40L, no IgM was detected (Fig 2E). CD40L alone resulted in approximately 200 ng IgM per well. By contrast, inclusion of IL-21 to CD40L-activated B cells resulted in maximal IgM production in the range of 3–4 μg per well (Fig 2E).

IL-4 was discovered as B cell stimulatory factor in the mouse and human [31, 32]. However, past work with both porcine and human IL-4 in a porcine splenocyte culture system showed that it elicited no stimulatory effect outside of acting as a potential maintenance factor for activated B cells [19]. In purified B cell cultures activated with CD40L we also found that IL-4 had no impact on proliferation (Fig 2B), on IgM production (Fig 2E), or on viability in the presence or absence of CD40L (Fig 2F).

### BAFF as a maintenance factor for porcine B cells

**Fig 3:**
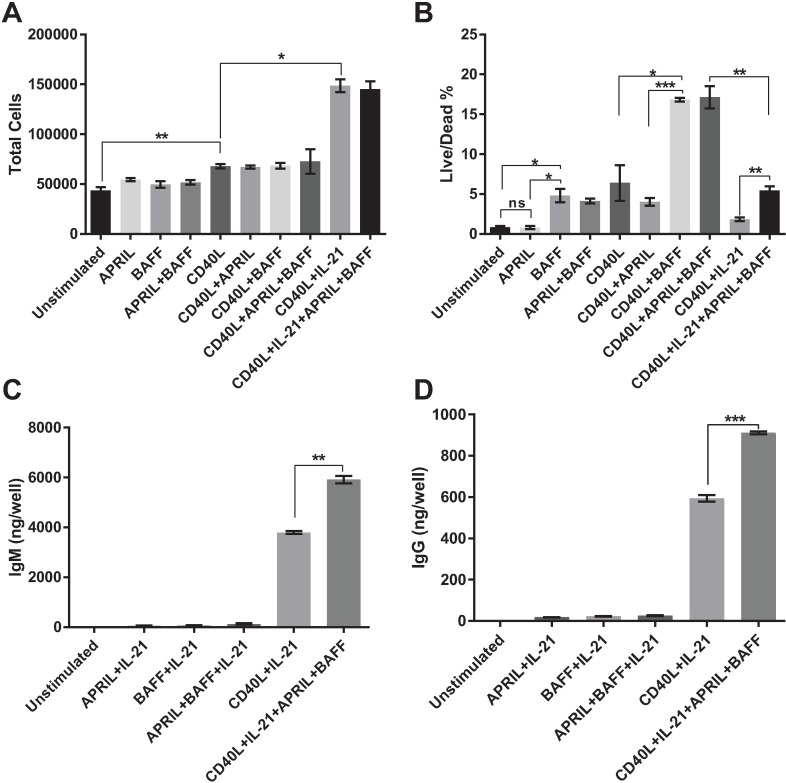
BAFF is a maintenance factor for porcine B cells. CD21+ purified B cells were treated with cytokines as described and cultured in technical triplicates. Culture supernatants and cells were collected and analyzed at day 7 for number of total cells via hemocytometer (A) and flow cytometry with fixable viability dye (B). Total secreted IgM (C) and IgG (D) were determined by ELISA as described in Materials and Methods. Results are representative of three independent experiments with three individual animals. Data are mean ± SEM. Statistical differences of *p < 0.05, ** p < 0.01, ***p < 0.001 were determined using ANOVA to compare group means followed by t-tests to calculate significance.

The decline of culture viability following IL-21 driven expansion, shown in Fig 2D, suggested that maintenance factors, such as BAFF and APRIL, might enhance cellular survival and function. BAFF and APRIL, members of the TNF family of ligands, are important for driving porcine B cell proliferation for certain subsets of B cells and ASCs [33, 34]. Treatment with BAFF and APRIL on non-activated, CD40L treated, and IL-21 stimulated cells did not result in an increase in cell numbers over seven days of culture, demonstrating that they did not affect B cell proliferation *in vitro* (Fig 3A). However, BAFF significantly enhanced cellular viability for both non-activated and CD40L activated B cells when compared to untreated cells and to cells treated with APRIL (Fig 3B). Treatment of cultures with both APRIL and BAFF did not result in significant differences in viability from treatment with BAFF alone. However, BAFF and APRIL together enhanced viability in cultures stimulated with CD40L and IL-21 (Fig 3B). As noted previously, IL-21 induced marked proliferation of B cells activated by CD40L binding. However, proliferating cells died rapidly, with a half-life of about 2 days (Fig 2D). BAFF and APRIL significantly increased proliferating cell viability, as shown in Fig 3B, with functional consequences of increased antibody secretion of both IgM and IgG (Fig 3C and 3D).

### BAFF and APRIL as maintenance factors for ASCs

**Fig 4:**
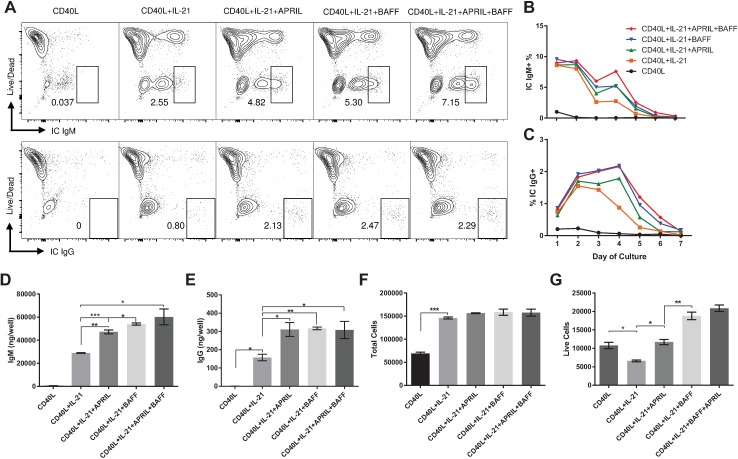
BAFF and APRIL support ASC viability. CD21^+^ B cells were cultured in triplicate in the indicated cytokines to determine the effects on IgM and IgG ASC differentiation at 4 days of culture (A). Cells were evaluated at 24 h intervals up to 7 days for live IC IgM^+^ (B) and IgG^+^ (C) cell percentages via flow cytometry, using the gating strategy and results for day 4 of culture displayed in panel A. On day 7, culture supernatants and cells were collected and analyzed for secreted IgM (D) and IgG (E) by ELISA. Total cells (F) were counted using a hemocytometer. Fixable viability dye and flow cytometry were used to determine cellular viability and then multiplied by total cell counts to calculate total live cell counts (G). Data (mean ± SEM) shown are from one of three equivalent experiments with three different animals. Statistical differences of *p < 0.05, ** p < 0.01, ***p < 0.001 were determined using ANOVA to compare group means followed by t-tests to calculate significance.

Although BAFF and APRIL did not affect B cell proliferation or differentiation, they have been shown to promote survival of ASCs in several species [1, 35, 36]. Therefore, we cultured CD40L activated CD21^+^ B cells with IL-21 and BAFF and APRIL and then evaluated cells every 24 hours for 7 days via flow cytometry for intracellular IgM and IgG in live cells. As shown in Fig 4A, the addition of IL-21 alone to CD40L activated cells resulted in the differentiation of approximately 8% of all B cells to IgM ASCs. There are two populations of live IC IgM+ cells displayed in Fig 4A. The IC IgM bright population is identified as IgM ASCs based on antibody production per well across treatments. The IC IgM dim population is likely a germinal center, non-antibody secreting, phenotype similar to that which has been observed in mice [37]. Time course analysis showed that IgM-positive cells were maximally induced within the first 24 hours of culture and BAFF and APRIL enabled a more sustained maintenance of positive cells (Fig 4B). IgG ASCs peaked at 48 hours of culture in CD40L activated and IL-21 differentiated cells, but the addition of APRIL and, especially, BAFF, maintained IgG-positive ASCs for up to 96 hours (Fig 4C), leading to a nearly two-fold increase in both IgM and IgG production compared to cultures without the maintenance factors (Fig 4D and 4E). BAFF appeared to be more effective in enhancing IgM production, as compared to APRIL, but there was no difference in IgG secretion (Fig 4D and 4E). No difference in total cell numbers was noted for any cultures treated with IL-21, ruling out APRIL or BAFF driven differences in antibody secretion due to effects on cellular proliferation (Fig 4F).

Lastly, culture viability was assessed using a fixable viability dye with flow cytometry and total cell counts at day 7 of culture. As shown previously in Fig 2D, IL-21 inclusion reduced total cell viability after 7 days compared to CD40L alone. However, APRIL restored IL-21-treated cultures to the level of CD40L alone and BAFF significantly enhanced live cell numbers (Fig 4G). Treatment with APRIL and BAFF together did not result in a statistically significant difference in viability over treatment with BAFF alone (Fig 4G). Overall, we have found that inclusion of both APRIL and BAFF on CD40L activated and IL-21 stimulated cells consistently results in numerical enhancement, which is not statistically significant, of cellular viability and antibody secretion as compared to treatment with either one alone.

### Maintenance factors inhibit ASC apoptosis

**Fig 5:**
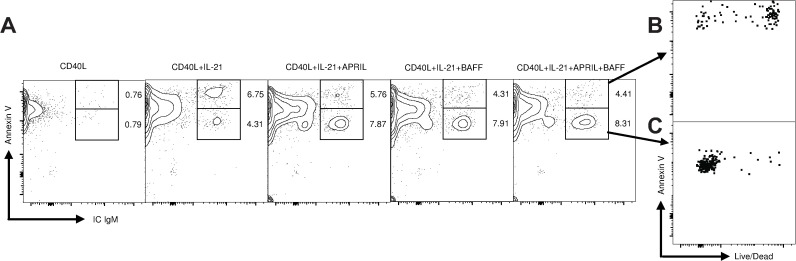
BAFF and APRIL extend ASC viability by delaying apoptosis. CD21^+^ cells were cultured in triplicate in the indicated cytokines. On day 4 of culture, cells were incubated with unlabeled anti-IgM, then stained for flow cytometry with a fixable viability dye, CD21, Annexin V, and then fixed and permeabilized and stained for IC IgM (A). Annexin V bright (B) and dim (C) populations were gated to show the live and dead distribution of the two populations. One representative experiment of two performed with two different animals is displayed.

The increase in ASC viability and antibody production observed with maintenance factor treatments led us to hypothesize that BAFF and APRIL were affecting cellular viability through the inhibition of ASC apoptosis. Hence, we labeled cells with a fixable viability dye, followed by annexin V staining, and then fixed and permeabilized the cells to detect intracellular (IC) IgM. IC IgM^+^ cells separated into two distinct populations displaying both bright and dim fluorescence associated with annexin V binding (Fig 5A). Gating on these populations showed that the majority of annexin V bright ASCs did not have intact plasma membranes (fixable viability dye, FVD^+^) and were thus considered non-viable (Fig 5B). The majority of annexin V dim cells maintained an intact plasma membrane (FVD^-^) and were still viable (Fig 5C). Treatment with IL-21, in the absence of a maintenance factor, resulted in elevated numbers of non-viable annexin V-bright ASCs and decreased numbers of viable annexin V dim cells as compared to IL-21 treated cultures which also received BAFF or APRIL, or both, as shown in Fig 5A. These results show that the positive effects of BAFF and APRIL on ASC viability and enhanced antibody production observed in Fig 4D and 4E are due at least in part to their ability to inhibit ASC apoptosis.

### IL-21 stimulates proliferation and differentiation of memory B cells

The identification of IC IgG+ cells at 48 hours of culture led us to hypothesize that IL-21 was inducing the proliferation and differentiation of activated memory B cells into ASCs. Thus, we evaluated the memory response of naïve and immune animals to PRRSV nsp7. The function of nsp7 is unknown, but it is highly immunogenic, thus increasing our opportunity to detect rare antigen specific memory B cells [3, 38]. Splenocytes were isolated from two non-exposed, negative and four immune mature female pigs that were infected with virulent field viruses >28 days prior to sacrifice. Cells were enriched for CD21, labeled with CFSE, and then treated with CD40L and the maintenance factors APRIL and BAFF to enhance ASC viability. IL-21 was added to half of the tissue culture wells and on day 3, at the peak of the *in vitro* IgG antibody response, equal aliquots of cells were used for proliferation analysis, transferred to PRRSV nsp7 and IgG ELISPOT plates, or were left in culture until day 14, when supernatants were harvested to test for nsp7-specific IgG by ELISA.

**Fig 6:**
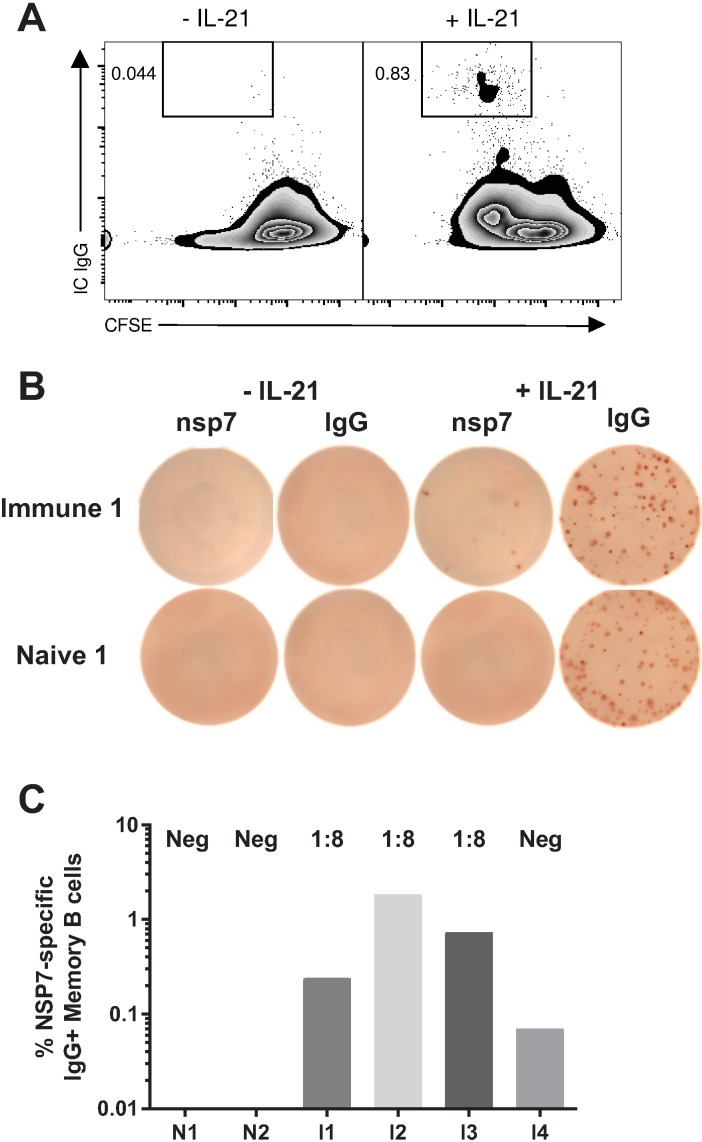
IL-21 proliferates and differentiates memory B cells. Splenocytes from four PRRSV immune and two naïve animals were enriched for CD21^+^ B cells. (A) Cells were labeled with CFSE and then cultured in the presence of CD40L, APRIL, and BAFF with or without IL-21. At day 3 of culture, 1/3 of cells were harvested and evaluated for IC IgG^+^ and CFSE divisions via flow cytometry. (B) PRRSV nsp7 (100,000 live cells/well) and IgG^+^ ELISPOT (10,000 live cells/well) were performed on 1/3 of cultured cells. Following normalization of plated cell number, the proportion of nsp7-specific spots to IgG^+^ spots was calculated and is displayed in (C). The remaining IL-21 treated cells were cultured for a total of 14 days. Supernatants were harvested and evaluated with a limiting dilution nsp7 ELISA. Individual titers are displayed above corresponding columns in (C). Data are representative of two independent experiments with the same animals.

To track proliferation, cells were analyzed by flow cytometry for cell division and IC IgG staining. B cells treated with IL-21 differentiated into IC IgG^+^ ASCs, but only after having undergone several rounds of division (Fig 6A). ELISPOT analysis showed equivalent numbers of IgG-secreting ASC in all animals after, but not before, stimulation with IL-21, and nsp7-specific antibody secreting cells only in immune animals (Fig 6B). Since the number of spots in the ELISPOT assay potentially exaggerated the proportion of IgG^+^ nsp7-specific memory B cells initially isolated from the spleen, all cultures were assayed both for nsp7-specific ASC differentiated from memory B cells and for IgG ASC to control for proliferation. All four PRRSV-immune pigs had memory B cells against nsp7, with more than a 20-fold difference between minimum and maximum frequency (Fig 6C). PRRSV naïve pigs were completely negative on nsp7 ELISPOTs. Antigen-specific ELISPOT results were supported by nsp7 ELISAs which showed three immune pigs with endpoint dilution titers of 1:8. Naïve control pigs and immune animal #4, with minimal ELISPOT values, showed background absorbance levels on ELISA (Fig 6C).

## Discussion

IL-21 plays a critical role in the adaptive immune response due to its ability to stimulate B cell differentiation and antibody production, and to promote the maladaptive development of inflammatory disease and autoimmune disorders, such as type 1 diabetes [39, 40]. However, limited data on IL-21 activities in the pig hinders progress in development of the pig for organ and islet xenotransplantation. It is of particular importance for type 1 diabetes, as IL-21 production is increased in type 1 diabetic patients, and resulting B cell involvement in disease progression seems likely [41, 42]. Furthermore, the cross-species activities of human CD40L and IL-21 on porcine B cells highlights the unanticipated host-graft interactions that may complicate xenotransplant acceptance.

Purified B cells placed into standard cell culture media die rapidly, indicating that their survival in the animal is dependent on cellular and molecular interactions in lymphoid tissues. CD40L activation by itself induces MHC II expression, makes cells permissive to IL-21, and increases cell half-life, but did not change B cell fate. IL-21 by itself had no apparent effect on survival, but in the presence of CD40L, it stimulated a robust proliferative differentiation of short-lived cells. The rapid decline in viability was consistent with an apoptosis inducing activity of IL-21, which has been previously described [43, 44]. In our hands, BAFF, but not APRIL, promoted resting B cell survival, suggesting that it was mediated through the BAFF-R as opposed to their shared receptors, transmembrane activator and CAML interactor (TACI) and B cell maturation antigen (BCMA). Both BAFF and APRIL were able to increase ASC viability and enhance production of both IgM and IgG by inhibiting apoptosis. These findings are consistent with other studies showing that BAFF/APRIL support plasmablasts derived from rapidly dividing memory B cells [1, 36, 45]. The specific receptor interactions mediating these effects may be dependent upon the type and location of the ASC [46–49].

Kinetic analysis showed that numbers of live IgM^+^ ASCs were highest within 24 h of B cell activation and stimulation and invariably declined after 48 h. IgG^+^ ASCs are a minor population that peaks at 48–96 h after stimulation, suggesting that they represent the memory B cell phenotype. This is quicker than has been observed in cultures of human class switched B cells from the spleen which peaked between days 3 and 4 of culture [2]. Comparison of nsp7-specific memory B cell ELISPOT and secreted antibody titers from the culture wells shows agreement supporting the conclusion that activated and proliferating memory B cells are the source of antibody production.

The lack of a stimulatory effect of IL-4 on porcine B cells is consistent with previous findings that IL-4 does not induce proliferation of porcine B cells [19], in stark contrast to its role in the mouse [50]. Additionally, its inability to affect B cell viability, as had been previously proposed, suggests that IL-4 may be unimportant in porcine B cell biology. We did not investigate if IL-4 may play a role in class switching to IgE, IgA, and subclasses of IgG [51–53].

The toolkit for defining B cell differentiation stages in the pig is smaller than in mice and humans. Antibodies for CD19 have not been characterized, and CD27 is not expressed on porcine B cells [54]. Therefore, we defined memory cells by CD21 positivity and antigen-specific functional analysis for PRRSV nsp7. The displayed ability of the T cell associated protein, CD40L, and IL-21, to proliferate and differentiate memory cells supports previous work showing that T cell help is necessary for in vitro activation and differentiation of memory B cells [1]. By using IL-21, along with other T cell and dendritic cell cytokines and factors, we were able to expand and differentiate memory B cells in the absence of potentially confounding secondary cell types and cytokines. Furthermore, the exhibited ability of IL-21 to elicit a robust memory B cell proliferation and differentiation response in the absence of antigen and BCR engagement is congruent with previous work which utilized mixed splenocyte-cultures [3]. These findings are consistent with previous results in human lymphocytes which first described the “bystander” effect and may be important for porcine maintenance of memory populations or autoimmune disease development [55, 56].

In conclusion, we demonstrate that IL-21 is a potent stimulator of CD40L activated porcine B cells, and that it is able to proliferate and differentiate rare memory B cells. It is now possible to address the molecular and cellular bases of immune response variation that confounds human and porcine vaccination efficacy using a relevant outbred species model.
